# Discretisation of conditions in decision rules induced for continuous data

**DOI:** 10.1371/journal.pone.0231788

**Published:** 2020-04-22

**Authors:** Urszula Stańczyk, Beata Zielosko, Grzegorz Baron

**Affiliations:** 1 Department of Graphics, Computer Vision and Digital Systems, Faculty of Automatic Control, Electronics And Computer Science, Silesian University of Technology, Gliwice, Poland; 2 Institute of Computer Science, University of Silesia in Katowice, Sosnowiec, Poland; Politechnika Krakowska im Tadeusza Kosciuszki, POLAND

## Abstract

Typically discretisation procedures are implemented as a part of initial pre-processing of data, before knowledge mining is employed. It means that conclusions and observations are based on reduced data, as usually by discretisation some information is discarded. The paper presents a different approach, with taking advantage of discretisation executed after data mining. In the described study firstly decision rules were induced from real-valued features. Secondly, data sets were discretised. Using categories found for attributes, in the third step conditions included in inferred rules were translated into discrete domain. The properties and performance of rule classifiers were tested in the domain of stylometric analysis of texts, where writing styles were defined through quantitative attributes of continuous nature. The performed experiments show that the proposed processing leads to sets of rules with significantly reduced sizes while maintaining quality of predictions, and allows to test many data discretisation methods at the acceptable computational costs.

## Introduction

Plenty of observed phenomena, objects, and problems are expressed through features that are real-valued. Such descriptions provide very detailed definitions of studied concepts. The available attributes and their values constitute a source of knowledge that can be used to construct a more general data model, which allows for pattern recognition and classification of unknown examples [1, 2].

Continuous nature of variables is not necessarily an advantage [3]. Firstly, quite simply, they are not always suitable for information systems as inputs. Secondly, they can be too specific to enable generalisation, thus limiting the predictive capabilities of the obtained data models, and possibly causing the risk of overfitting. Due to the applied representations, the real-valued features and built on them systems can also have significantly higher requirements for storage and processing time.

Therefore, typically a tough decision needs to be made, either to accept continuous values of attributes with all consequences, or, to implement some of discretisation algorithms [4]. They attempt to simplify and reduce data by transforming continuous input space into granular and discrete one, grouping occurring values into some number of ranges called bins. Categorical representation is more concise and by that more general, and allows for more compact forms of inducers. Discretisation most often involves some loss of information, and this fact works both as an advantage and a disadvantage. When discretisation procedures are a part of input data pre-processing, preceding data mining, then we mine data from which some information was irrevocably removed. The differences in patterns, clearly detectable in continuous domain, can become blurred or even completely obscured in discrete domain, and data models learned from such data can suffer as a result. What is more, due to the high number of existing discretisation methods, the choice of an algorithm that would be best suited to any given problem, is not a trivial task [5].

In an ideal scenario, at the stage of data mining we would appreciate access to all available information, which means operating on real-valued features. Once all knowledge about described concepts is discovered, we would like it to be represented in such form that discards all unimportant, unnecessary details, and keeps broader general categories, which can be obtained through discretisation. To achieve these two aims the paper proposes a new approach, which can be applied to inducers capable of working with both continuous and nominal attributes, and allowing for easy access to structures representing learned knowledge, such as rule classifiers. Decision rules store information about patterns detected in data by listing conditions for features that lead to class labels. This transparent form enhances understanding, and is one of the reasons why rule classification systems are often preferred as inducers [6].

Studied decision rules were induced within Dominance-Based Rough Set Approach (DRSA) [7], which is a modification of Classical Rough Set Approach (CRSA). Rough set theory, firstly proposed by Z. Pawlak [8], works well in tasks with incomplete and uncertain data [9]. DRSA allows for nominal as well as ordinal classification due to observing orderings in value sets, and replacing indiscernibility (fundamental for CRSA) with dominance relation. It is often employed for multi-criteria decision making.

In the proposed methodology firstly decision rules were inferred from input continuous data. Secondly, data was discretised by various approaches. Applying definitions of categories obtained for all features, in the third step conditions in previously induced rules were discretised, translating decision algorithms from real into discrete space. And finally, the characteristics of discretised systems of rules, such as coverage and reduction of storage requirements, were studied, while performance was evaluated by application of rule classifiers to independently discretised test sets [10, 11]. With this new approach several discretisation methods could be considered for a task at a lower cost. Typically the hardest step of knowledge induction was executed just once, and only discretisation procedures were performed repeatedly. They were markedly less demanding of computational resources.

In this research framework, sets of decision rules were also induced from discrete data. Their power was compared to discretised algorithms, and the original versions operating in continuous domain. The results from the experiments show that the changed scenario of data processing is worth of considerations, both with respect to performance of constructed classification systems and size reduction of their structures. The obtained results allowed also for observations on interesting differences in trends visible between applied discretisation methods.

The patterns, detected and classified by generated decision algorithms, reflected characteristic properties of writing styles. These stylometric features provided descriptions for linguistic preferences of authors in a numerical form [12]. It enabled authorship attribution of texts with unknown or disputed authorship [13, 14] to be executed as a classification task, with stylometry as an application domain. In the presented study the input data sets were prepared for binary classification with balanced classes.

The paper is organised in the following manner. Section Background and related works explains motivation leading to research and presents theoretical background, with descriptions of rough set processing applied to data, specifics of characteristic features in stylometric domain, and discretisation approaches. Section Setting up the experiment provides the details of research framework, while Section Observations on research results includes specific comments to the obtained results. Section Concluding remarks contains the summary and conclusions, and indicates directions for future research.

## Background and related works

The section presents formulation of the problem and motivation for research, which is followed with the theoretical background. There are given brief descriptions for all involved areas, namely rough set processing as a way to mine data, rule induction algorithms, stylometric analysis of texts, and selected approaches to discretisation of input continuous data.

### Problem formulation and motivation

The motivation for the new methodology proposed in the paper originated in the observation that in many application areas analysed and studied concepts are described with continuous characteristic features, which are discretised in order to facilitate representation of information and the process of data mining. As the notion of discretisation has been studied for years, there are many categories of algorithms to choose from, and various methods were employed in research works [4, 15].

While some data mining methods require discrete data, there are also some techniques capable of reasoning based on real-valued attributes, for example Naive Bayes, Artificial Neural Networks [16], or decision trees. Among the latter C4.5 and C5.0 are popular algorithms [17, 18], which handle both continuous and discrete attributes. In [19], the Authors proposed a hybrid technique for data classification, and showed that neural networks were better than the direct application of induction trees in modelling nonlinear characteristics of raw data.

There were also conducted studies where inducers handled both types of data, but could perform better with discretised features [20–22]. Performance of Naive Bayes classifiers in continuous and discrete domain was studied e.g., in [10, 23]. The Authors of [24] compared classification accuracy for raw and discretised data, and showed that discretisation helped to improve the performance of Naive Bayes model.

In [5], an influence of discretisation methods on the power of associative classifiers was investigated. Experimental results indicated that the performance of classifiers significantly varied with the change of employed data discretisation algorithm.

Decision rule classifiers also belong with data mining approaches that allow to learn knowledge from continuous data. They can be based on a decision tree, or obtained directly from the training data using different rule induction algorithms. They are often preferred as inducers, because they offer also easily accessible and clear representation of discovered knowledge, which enhances understanding of learned patterns [6, 25].

In [26], the Authors compared the classification accuracy of rule sets induced after discretisation of data based on conditional entropy, with those inferred by MODLEM algorithm, where discretisation and rule induction were executed at the same time. The results did not display significant differences. In [27], MLEM2 algorithm was proposed for rule induction from numerical attributes. The obtained results showed that discretisation performed simultaneously with rule induction caused better classification accuracy of rule classifiers than in case of discretisation preformed as a pre-proceesing data step. Moreover, the size of rule sets and the total number of conditions were smaller in case of MLEM2 algorithm.

As there are many discretisation procedures, both parametric and non-parametric [28], the observation that the power of a classification system is highly dependent on the selection of a discretisation method, brings the conclusion that a choice of the algorithm that would be best suited for any given task is not straightforward.

One of possible attempts at finding a solution to this problem, is to perform discretisation by several procedures, with varying parameters, and execute data mining for all versions of discrete input data sets. Then, by evaluation of performance of obtained classification systems, we could choose the best variant. However, such methodology could be unfeasible, as it would require repeating the knowledge discovery process multiple times, and this step often is the most time consuming part of the entire data mining task, with the highest computational demands and costs.

Following this line of reasoning, in the paper a new methodology was proposed. Its originality lies with the reversed order of processing steps, where knowledge discovery (induction of decision rules) precedes discretisation, and not the other way round, as in other traditional approaches. The novelty of this research framework comes down to discretisation of not just data but also the learned knowledge, captured in the directly accessible conditions on attributes, included in the premises of inferred rules.

In the first step of processing, decision rules are induced from continuous input data sets. Secondly, all input sets are independently discretised with various methods. Employing definitions of intervals constructed for all features in discretisation of training sets, in the third step, the real-valued conditions in rules are replaced with their categorical representations. It results in obtaining sets of discrete decision rules with greatly decreased sizes.

The proposed methodology allows to try many discretisation algorithms for data, with the acceptable overall computational costs. The step of rule induction, which typically is the most demanding, is executed only once, and only discretisation is performed several times. Even though it is more complex, as not only input data sets are transformed but constructed rule sets as well, still the process requires less time and effort than it would take in the traditional experimental setting. This increased complexity of discretisation can be considered as a disadvantage of the methodology. Also, the possibility that for some variant of discrete data better rules could be inferred cannot be entirely ruled out. Yet the guarantee of choosing the best discrete representation of data could only be found in exhaustive induction and comparison of rule sets from all possible versions of discrete data sets.

### CRSA vs. DRSA

Rough set processing is dedicated to cases of uncertain, imprecise, and incomplete knowledge [3, 9]. In set theory only crisp sets are distinguished, and elements are either not included or included in some set. In Classical Rough Set Approach, as originally defined by Pawlak [25], not only elements that are included in a set are defined, but also elements that could belong to the set. And further, in Dominance-Based Rough Set Approach there are recognised elements which can be included at most, or at least in some set [7, 29]. Thus inherent mechanisms of rough processing allow to define imprecise concepts through approximations, imposing a specific granular perspective upon the universe of discourse.

The available information about the universe is stored in the form of *decision tables*. A decision table consists of a finite set of objects of the universe *U*, a finite set of condition attributes *A* = {*a*_1_, …, *a*_*n*_}, and a finite set of decision attributes *D*. In multi-criteria decision making condition attributes are called criteria, and it often happens that there is a single decision attribute *D* = {*d*}. It partitions the universe into a finite number of recognised classes ***Cl*** = {*Cl*_*t*_}, with *t* = 1, …, *m*.

CRSA works only for categorical attributes, as the universe of discourse is perceived through granules of knowledge corresponding to *equivalence* classes of objects that cannot be discerned by the values of their attributes. With the *indiscernibility* principle, CRSA argues that when two objects *x* and *y* of the universe cannot be discerned, they should be classified in the same way—that is to the same class. In consequence CRSA is capable only of observing presence or absence of some property, which leads to nominal classification.

In DRSA *dominance* relation replaces indiscernibility, and values in all input data sets are perceived as ordered. Let ≽_*q*_ be a relation of weak preference defined for the set of objects with respect to a criterion *q*. Let *I* = {1, …, *n*} be a set of the indices of condition attributes and, without a loss of generality, $f_{a_{i}}:U\to R$, for each *i* ∈ *I*. For all objects *x*, *y* ∈ *U*, $f_{a_{i}}\left(x\right)\geq f_{a_{i}}\left(y\right)$ means that “*x* is at least as good as *y* with respect to attribute *a*_*i*_”, and it is denoted as $x{≽}_{a_{i}}y$. It is said that *x* dominates *y* with respect to a set of attributes *P* ⊆ *A*, when the values of all considered attributes for *x* are at least as good as their correspondents of *y*, that is if for every attribute *a*_*i*_ ∈ *P*, $f_{a_{i}}\left(x\right)\geq f_{a_{i}}\left(y\right)$. It is denoted by *xD*_*P*_ *y*. The relation of *P* *-dominance* is reflexive and transitive. *y* is *dominated* by *x* when all attributes of *y* considered as criteria have values at most as good as those of *x*. Respectively we have a set of objects dominating *x*, and a set of objects dominated by *x*. These sets correspond to granules of knowledge recognised in DRSA:

- *P*-dominating set—a set of objects dominating *x*:
  $D_{P}^{+}\left(x\right)=\left\{y\in U:yD_{P}x\right\}$,
- *P*-dominated set—a set of objects dominated by *x*:
$D_{P}^{-}\left(x\right)=\left\{y\in U:xD_{P}y\right\}$.

The dominance or *Pareto* principle states that when an object *x* is as good as another object *y* (with respect to values of the attributes considered as criteria), then *x* needs to be classified *at least as* good as *y*. On the other hand, if *x* is not better than *y* with respect to their attributes, *x* should be classified *at most as* good as *y*.

Decision classes are preference ordered (as any other values of attributes in DRSA), with increasing indices leading to increased preferences, that is for all *r*, *s* ∈ {1, …, *m*}, such that *r* > *s*, the objects from *Cl*_*r*_ are preferred to the objects from *Cl*_*s*_. Where no clear natural order in data exists, it needs to be defined or discovered [30]. An attribute is considered as *cost* type when its lower value is preferred over higher, and for *gain* type higher values are preferred over lower ones. Decision classes are always considered as gain. For condition attributes of cost type lowest values point to more preferred classes, while gain type links higher values of criteria with more preferred classes.

The upward or downward unions of classes are called *dominance cones*, Eq (1).

$$\begin{array}{c} Cl_{t}^{\geq }=\underset{s\geq t}{⋃}Cl_{s},\;\;Cl_{t}^{\leq }=\underset{s\leq t}{⋃}Cl_{s},\;\;t=1,\ldots ,m. \end{array}$$ (1)

*P*-lower approximation of $Cl_{t}^{\geq }$, for *P* ⊆ *A*, is the set of objects that belong to $Cl_{t}^{\geq }$ without any ambiguity. It is denoted as $\underline{P}\left(Cl_{t}^{\geq }\right)$. *P*-upper approximation of $Cl_{t}^{\geq }$, denoted as $\bar{P}\left(Cl_{t}^{\geq }\right)$, is the set of objects that could belong to $Cl_{t}^{\geq }$,

$$\begin{array}{c} \underline{P}\left(Cl_{t}^{\geq }\right)=\left\{x\in U:D_{P}^{+}\subseteq Cl_{t}^{\geq }\right\}, \end{array}$$ (2)

$$\begin{array}{c} \bar{P}\left(Cl_{t}^{\geq }\right)=\left\{x\in U:D_{P}^{-}\cap Cl_{t}^{\geq }\neq \emptyset \right\}, \end{array}$$ (3)

Calculation of approximations for dominance cones is the starting point for the process of induction of decision rules. These rules provide conditions on some attributes *a*_*i*_ and their values $v_{a_{i}}$ and can take four general forms.

$$\begin{array}{c} if\;\left(a_{1}\leq v_{a_{1}}\right)\wedge \ldots \wedge \left(a_{n}\leq v_{a_{n}}\right)\;then\;Cl_{t}^{\leq } \end{array}$$ (4)

$$\begin{array}{c} if\;\left(a_{1}\geq v_{a_{1}}\right)\wedge \ldots \wedge \left(a_{n}\geq v_{a_{n}}\right)\;then\;Cl_{t}^{\geq } \end{array}$$ (5)

$$\begin{array}{c} if\;\left(a_{1}\geq v_{a_{1}}\right)\wedge \ldots \wedge \left(a_{n}\geq v_{a_{n}}\right)\;then\;Cl_{t}^{\leq } \end{array}$$ (6)

$$\begin{array}{c} if\;\left(a_{1}\leq v_{a_{1}}\right)\wedge \ldots \wedge \left(a_{n}\leq v_{a_{n}}\right)\;then\;Cl_{t}^{\geq } \end{array}$$ (7)

Forms (4) and (5) are induced for attributes of gain type, and forms (6) and (7) for cost. If types of attributes are not uniform, and some are gain while others cost, then within a rule there is a corresponding combination of forms of conditions. The rule classifies to either at most some class by assigning $x\in Cl_{t}^{\leq }$ (as in Eqs (4) and (6)), or at least some class for $x\in Cl_{t}^{\geq }$ (as in Eqs (4) and (6)). As a result, DRSA does not only observe the presence or absence of a property, but also monotonic relationships within data, evidenced by preference orders in the description of objects by condition and decision attributes. It leads to not only nominal, but also ordinal classification [31].

DRSA processing does not require any prior discretisation of real-valued attributes, only definitions of preferences. It means that constructed decision algorithms can operate directly in continuous domain. When conditions included in rules refer to continuous values, they provide very close descriptions for recognised concepts. Yet such precision comes at a cost of reduced generality, possibly more complex calculations, and prolonged processing. Thus any chance at simplification or improvement gives motivation for research. It was of sufficient interest to observe how categorical values of attributes obtained through various discretisation approaches reflect on rule classifier performance to warrant experiments presented in the paper.

### Rule induction algorithms

Decision algorithms are often favoured as inducers. Due to their transparent structure, they directly indicate conditions that need to be satisfied to associate a sample with a class [32, 33]. When applying decision rules for classification of examples, we often refer to notions of support and coverage. Support of a rule indicates for how many objects in train data the rule is true (both the premise and the conclusion parts of the rule need to be true), or, in other words, how many learning instances support this rule. Coverage of the rule indicates for how many objects in a data set the premise part of the rule is true. In the case of application to train data, these two terms can be used interchangeably, as no rule should incorrectly classify a learning example—when coverage exists, also support follows. In the case of evaluation and test instances, a rule can show coverage without providing correct classification, which then causes either false positive or false negative.

There are many classification strategies based on decision rules [34]. Taking into account ordering of rules, they can be divided into two groups: classifying with lists of rules (rules are applied successively and ordering of rules is significant), and sets of rules (rules are applied simultaneously and ordering of rules is insignificant). While focusing on inferred decision rules, we can observe: algorithms inducing minimal sets of rules, algorithms inducing exhaustive sets of rules, and algorithms inducing satisfactory sets of rules (where satisfactory means meeting some criteria defined by a user) [9, 35].

Minimal cover approach follows the general heuristic strategy, which is used for many known machine learning algorithms e.g., AQ [36], CN2 [37]. It is based on constructing a first rule by sequentially choosing these elementary conditions which are the “best” according to some heuristic criteria. Examples that match this rule are removed from the learning set. The process is repeated iteratively as long as some learning examples remain uncovered, and stopped as soon as the obtained set of rules covers all train instances. Within the research described in the paper the minimal cover rule sets were inferred with DOMLEM algorithm implemented in 4eMka software [29]. Rules induced with minimal cover approach are found relatively quickly, and cardinalities of rule sets are low. It results in fast processing, which is an advantage of this approach and the reason for preference for such algorithm. On the other hand, there is no guarantee that the best rules are discovered.

Exhaustive rule induction algorithm constructs all minimal rules that can be generated for given examples [1]. It allows to obtain the richest information about all patterns existing in the analysed data set, not only those that are most often repeated. Among created rules there are ones which correspond to the same or overlapping sets of objects, and rules having similar condition parts. In consequence, the number of rules inferred in exhaustive search typically is significantly higher than the number of rules induced by minimal cover approach. Exhaustive approach is the most expensive from the point of view of processing time, computational complexity, and storage requirements. However, several examples can be found, where this approach was successfully applied in classification, either directly or with using some additional processing e.g., rule filtering for the purpose of increased performance [6, 38, 39]. High costs speak against exhaustive algorithms, but, contrary to minimal cover, they do guarantee to include decision rules with the highest quality [40], as literally all rules on examples are induced. This type of algorithm was also used in the research.

### Stylometric characteristic features

Analysis of texts leading to tasks of author characterisation (or profiling) and authorship attribution (or verification), is a prominent field of study, with probably the most influential early works due to Mosteller and Wallace [41]. It attracts more and more attention with the constant increase of textual data sent, stored, and processed for various purposes [12]. Studied text samples vary in considered lengths, type of media, employed linguistic registers, and many other properties. Along with myriads of intended applications, it causes a wide range of stylometric markers to be used as characteristic features, describing writing styles in quantitative terms [42]. These style descriptors can be put into many categories, for example content-specific, structural, lexical, or syntactic type [43].

For content-specific markers, their definitions depend on a subject topic, as they refer to words of a certain meaning in a context (key words). Structural markers are used to describe specific formatting or organisation of text elements. In the case of digital media, they include font types, embedded links or pictures, emoticons, and for a hard copy also page layout, or handwritten or drawn elements can be considered. These two types, together with language-specific descriptors, are also referred to as application-specific features.

Lexical features base on employed vocabulary with its richness, and statistical information on linguistic elements present in a text sample. They can specify averages, frequencies of occurrence [44], or distributions of selected elements [45], which explains their inherent continuous nature. Inside this group, character features are distinguished, which refer to single characters (letters and digits) or their groups, of either fixed or varied length (but not necessarily forming words [46]). When synonyms and semantic dependencies are studied, with considerations of parts of speech, the resulting style markers can be treated as lexical, placed among syntactic descriptors, or form a separate category of semantic markers.

Syntactic descriptors capture the underlaying patterns in constructed structures of phrases and statements, as evidenced by complexity of sentences, application of passive voice, or included punctuation marks [47].

A group of features that allow for a unique definition and recognition of an author based on their style, is referred to as a *writer print* or *author print*. As with any other prints we leave behind, there can be more than one such set. A textual analysis with respect to writing styles relies on finding such characteristic features, which form an author-specific base of attributes. Styles are defined by quantitative descriptors referring to such linguistic elements that are used rather semi-consciously or subconsciously. They are hidden inside habitually preferred phrases, in favoured patterns of formulated sentences [48]. They should enable authorship attribution with sufficient level of reliability, regardless of a subject content of a text, and despite possible style variations appearing when compared text samples were written even years apart [49]. The latter problem can be studied as a task with so-called concept drift [50]. In order to execute attribution we need to compare a text sample of unknown or questioned authorship with others which are reliably attributed. It means looking for a solution for a supervised learning task.

Methods and techniques employed in authorship attribution studies often use some statistics-oriented computations, for example by constructing language models based on the probabilities of occurrence for the chosen sequences of characters, words, or phrases [51]. Another way of processing is adaptation and application of some data mining approaches [52, 53], for example Artificial Neural Networks [16], or rule classifiers [35].

Reliability of style markers and author recognition depends also on the construction of input data sets, obtained samples, and their groups. Descriptors need to be calculated over text samples of comparable and sufficient size, neither too large nor too small [54]. The former limits the number of available examples and obscures some style variations that can be useful in discrimination, while the latter returns strictly local and detailed characteristics, which can be hard to find in other texts.

Comparable sizes of text samples typically imply that larger works are divided into different numbers of smaller parts. In such examples that are based on various parts of the same longer text, similarities can be detected for obtained characteristics, closer than when compared to other samples, based on separate texts. This observation leads to an important conclusion. When authorship attribution is treated and executed as a classification task, in order to evaluate classifier performance we need to use evaluation and test sets, and not popularly employed cross-validation. In cross-validation, even with several folds, it is highly probable to obtain falsely higher classification accuracy. These overly optimistic results are explained by this close similarity of some groups of examples [55], and lack of statistical independence between tests, as the same samples are used in several evaluations [11].

Evaluation and test sets need to use samples based on works which are separated from training. This independence of input sets gives higher reliability of obtained results. However, when discretisation approaches are employed to these sets, independently executed procedures (which is the simplest way) can result in additional problems with varying numbers of intervals defined for the same variables in different sets [10].

### Discretisation approaches

Data pre-processing is one of the important steps preceding the execution of data mining [1]. It consists of: (i) data cleaning methods, which can be used for removal of inconsistencies and noise contained in the data, (ii) data transformation methods, which can improve the efficiency of distance-based mining algorithms, and (iii) data reduction methods, which allow to obtain such representation for a data set that is reduced in volume but produces either the same or very close analytical results. All pre-processing methods are important for knowledge discovery, because they aim to improve the quality of the data, and help to successfully execute data mining tasks.

One of data reduction mechanisms is discretisation. It transforms numerical real-valued attributes into discrete or nominal ones with a finite number of intervals [15], by mapping input continuous space of attributes’ values into a reduced subset of their categorical representations.

Discretisation process simplifies the data and removes possible noise from them. Thanks to this, the data is easier to understand, interpret and use. One of disadvantages of discretisation is that it causes some loss of information. Which is why discretisation process should always be executed with caution, and adjusted to the available data.

There are many discretisation methods and approaches, and they can be divided using various criteria [22]. Below popular categorisations can be found.

- Supervised vs. unsupervised;
  If a discretiser takes into account class information for samples to find the proper intervals among the ranges of the attribute values, it is called supervised. It uses some heuristic measures, e.g., entropy, to determine the best out of candidate cut-points. An unsupervised discretiser does not consider class information and the number of constructed intervals is provided as an input parameter.
- Static vs. dynamic;
  A static discretiser is independent from the learning algorithm, and performs transformation of attributes before the learning task. Dynamic discretisers are the components of the learning algorithm, and they are based on the information exchange between the discretiser and learner units. Most of discretising methods are static, dynamic discretisers are considered to be a part of data mining approaches.
- Univariate vs. multivariate;
  A univariate discretiser works with a single attribute at a time, contrary to a multivariate one, which bases on the interactions among the attributes, and simultaneously considers values of all attributes to define the set of cut-points.
- Local vs. global;
  The work of local discretisers is limited to some distinctive parts of the attribute domain, for which they are defined separately. To obtain the initial set of cut-points, a global discretiser analyses the full ranges of values for the attributes.
- Top-down vs. bottom-up;
  Top-down discretisers initially assign one large interval to represent all known values of an attribute, and then this interval is partitioned into some number of smaller and smaller sub-intervals, until a certain stopping criterion is met. In a bottom-up approach, the processing starts with some number of intervals determined by the set of cut-points, and during execution these intervals are combined together, until a certain stopping criterion is achieved.

In general, discretisation can be considered as a four-step process [22]:

1. sorting all values of a discretised attribute, in either descending or ascending order;
2. establishing the cut-points (for splitting intervals, or for merging intervals);
3. splitting or merging intervals, according to an algorithm criterion.
  In a top-down approach, intervals are split, in a bottom-up approach intervals are merged. For splitting, all candidate cut-points are evaluated, the best one is chosen, and it splits the considered range of continuous values into two bins. Discretisation is continued with each interval until a stopping criterion is achieved. Similarly for merging, neighbouring bins are evaluated to find the best pair to merge in each iteration. Discretisation continues with the reduced number of intervals as long as a stopping condition remains unsatisfied.
4. stopping the discretisation process, which depends on a specific criterion defined in the discretisation algorithm.

#### Unsupervised discretisation methods

The two most popular unsupervised discretisation algorithms are equal width binning and equal frequency binning [4]. The former sorts the values of a discretised continuous attribute, designates the minimum and maximum values of the attribute, and then divides the range into *k* disjoint discrete intervals with equal width, where *k* is an input parameter defined by a user.

Equal frequency binning also finds the minimum and maximum values of the discretised attribute, sorts all values in an ascending order, and divides the resulting range into a number of intervals defined by a user, so each bin contains the same number of sorted values [28]. For this method, repeated occurrences of the same continuous value could cause that such value would be assigned into different bins. During construction of the cut-points, it is important that any duplicated values are detected and directed always to one and the same bin, even if it means unequal numbers of occurrences in established intervals, or smaller than the requested number of bins.

The two methods are simple (which works to advantage), and sensitive with respect to a number of bins defined by a user (which could become a disadvantage). One of drawbacks is that in cases where the values of a continuous attribute are not distributed evenly, some information can be lost after the discretisation process.

There is a modification of equal width binning algorithm that is based on leave-one-out estimation of entropy. The obtained numbers of bins and cut-points allow to construct the discrete data set, which better reflects the nature of the input data. This optimised version of the algorithm (as implemented in WEKA environment [2], used in the research) contains three steps:

1. ∀*a* ∈ *A* the distribution table is calculated, where
  *A*—the set of attributes,
  *b* = 1…*B*, *B*—maximum number of bins defined by a user,
  *d*(*a*, *b*)—the number of instances of the attribute *a* in each bin for given *b*.
2. Entropy for all attributes is estimated by the following calculation:
  $$\begin{array}{c} b_{opt}\left(a\right)=\underset{b}{\mathrm{argmin}}H\left(a\right)=-\sum_{k=1}^{b}d\left(a,k\right)\log\frac{d(a,k)-1}{w(a,k)}, \end{array}$$ (8)
  where *w*(*a*, *k*) is the width of the bin for the given attribute *a*, and *k* is the number of bins.
3. ∀*a* ∈ *A* cut-points for *b*_*opt*_(*a*) are calculated.

An alternative version exists also for equal frequency binning algorithm. Instead of requiring the number of bins, the weight of instances per bin is set as the input parameter. When the instances are not weighted, this processing delivers such resulting number of bins that contain the assumed number of instances.

Due to the definition of constructed intervals provided by the user, the described unsupervised discretisation mechanisms in their standard versions do not cause significant problems when several separate sets of samples need to be discretised. To simplify transformations, these sets can be processed independently. It results in constructing independent discrete data models, which are then compared within the classification process. With this independent processing, the ranges of the intervals, established for the same attributes in different sets, can vary to some extent. Yet the numbers of these intervals will be as required.

#### Supervised discretisation methods

Two most popular methods from supervised category were proposed respectively by Fayyad and Irani [56], and Kononenko [57]. They base on class entropy of the considered intervals for evaluating candidate cut-points, and Minimum Description Length (MDL) principle as a stopping criterion.

Let set *S* contain *N* instances and *t* decision classes *Cl*_1_, …, *Cl*_*t*_. Class entropy *H*(*S*) of *S* is defined as follows:

$$\begin{array}{c} H\left(S\right)=-\sum_{i=1}^{t}P\left(Cl_{i},S\right)\log\left(P\left(Cl_{i},S\right)\right), \end{array}$$ (9)

where *P*(*Cl*_*i*_, *S*) is the proportion of class *Cl*_*i*_ instances in *S*.

Taking into account binary discretisation of a continuous attribute *a*, the optimal selection of cut-point *T*_*opt*_ is made by testing all possible candidate cut-points *T*. A cut-point *T* splits set *S* into two subsets, *S*_1_ and *S*_2_, where *S*_1_ ⊆ *S* contains the instances with the attribute values ≤*T*, and *S*_2_ = *S*\*S*_1_. Then entropy for the cut-point *T* is calculated as:

$$\begin{array}{c} H\left(a,T;S\right)=\frac{|S_{1}|}{\left|S\right|}H\left(S_{1}\right)+\frac{|S_{2}|}{\left|S\right|}H\left(S_{2}\right). \end{array}$$ (10)

For the optimal cut-point *T*_*opt*_ class information entropy *H*(*a*, *T*_*opt*_;*S*) is minimal.

The process of finding cut-points is top-down: it starts with considering one interval including all occurring values of a discretised attribute. Then its partitioning is repeated in a recursive way, until a stopping criterion is met. It is possible that discretisation results in all values of some attribute being grouped into a single interval. It happens when the calculated entropy indicates that this particular attribute does not contribute to discrimination of classes within the confines of the particular set of instances. For the same set for another variable several intervals can be required. Following this line of reasoning, supervised discretisation can be considered a process of knowledge discovery. Information about the numbers of bins constructed for each variable can be treated as a new source of available knowledge, to be mined and used for other purposes [58, 59].

Contrary to unsupervised discretisation methods, for supervised discretisation there can be some serious consequences of independent processing for the input data sets. Not only the cut-points can be different, but, which is far more problematic, their numbers and the resulting from them numbers of the constructed intervals can greatly vary, depending on the local context of each transformed set [60]. It can lead to obtaining discrete data models so dissimilar that it makes the classification task more difficult.

#### Fayyad and Irani MDL

In the case of Fayyad and Irani method, the stopping criterion for the top-down process of evaluating cut-points is connected with information gain—the difference in entropy without and after splitting the interval. Discretisation is applied as long as information gain, resulting from accepting the cut-point *T*, exceeds the value based on MDL principle and defined in Eq (11):

$$\begin{array}{c} Gain\left(a,T;S\right)=H\left(S\right)-H\left(a,T;S\right)>\frac{\log_{2}\left(N-1\right)}{N}+\frac{\Delta (a,T;S)}{N}, \end{array}$$ (11)

where

$$\begin{array}{c} \Delta \left(a,T;S\right)=\log_{2}\left(3^{t}-2\right)-[t\cdot H\left(S\right)-t_{1}\cdot H\left(S_{1}\right)-t_{2}\cdot H\left(S_{2}\right)]. \end{array}$$ (12)

*t*_1_ and *t*_2_ denote respectively the numbers of classes represented in the subintervals *S*_1_ and *S*_2_.

#### Kononenko MDL

In the case of Kononenko method, the process of discretisation is applied recursively until the following inequality (13) is satisfied:

log(NNCl1…NClt)+log(N+t-1t-1)>∑jlog(NajNCl1aj…NCltaj)+∑j(Naj+t-1t-1)+logNT, (13)

where

$N_{Cl_{i}}$—the number of training instances from the class *Cl*_*i*_,

$N_{a_{x}}$—the number of instances with *x*-th value of the given attribute *a*,

$N_{Cl_{i}a_{y}}$—the number of instances from class *Cl*_*i*_ with *y*-th value of the given attribute *a*,

*N*_*T*_—the number of candidate cut-points.

## Setting up the experiment

**Fig 1:**
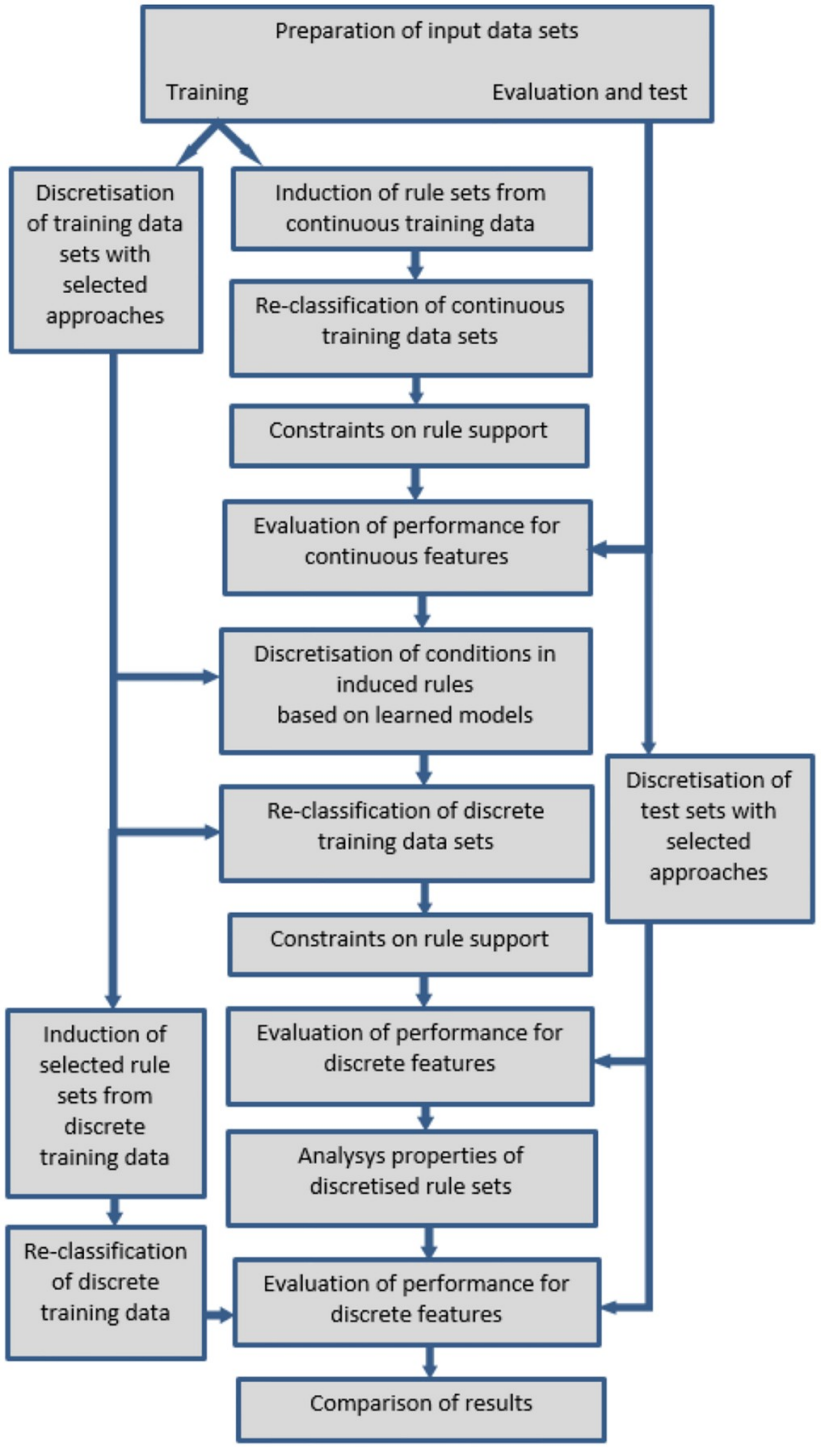
The procedure of executed experiments.

Executed experiments included preparation of the input data sets, induction of decision rules in continuous domain, discretisation of data sets and rule sets, induction of decision rules in discrete domain, evaluation of performance for all constructed classifiers, and analysis of all experimental results. The procedure is illustrated in a diagram given in Fig 1, and explained in the following subsections.

### Proposed research framework

The proposed methodology consisted of the steps as follows:

1. initial data processing
  - defining the task of authorship attribution by choosing writers for comparison,
  - selecting specific works for textual analysis,
  - constructing text samples,
  - choosing stylometric features and obtaining their values for all samples;
2. rough data mining with DRSA approach for continuous data
  - induction of decision rules with exhaustive and minimal cover algorithms,
  - re-classification of training samples for the purpose of establishing hard constraints on rule supports,
  - evaluation of performance for test sets;
3. discretisation through various methods for
  - all input data sets,
  - conditions in the previously inferred decision rules;
4. rough data mining for discrete data
  - induction of decision rule sets with minimal cover algorithm,
  - re-classification of training samples with discretised and discrete rule sets to obtain hard constraints on rule supports,
  - evaluation of performance for test sets;
5. analysis of results
  - properties of discretised and discrete rule sets,
  - observations on trends in performance for classifiers,
  - conclusions from comparisons of obtained results.

All steps are described in detail in the following subsections, and discussion of the experimental results is provided in the next section.

### Input data sets

To achieve a fair comparison of writing styles, considered authors need to come from some similar time period. Otherwise, the linguistic differences resulting from the constant evolution of any used language could be far too striking. Furthermore, to obtain reliable characteristics, for every author many texts are required, each of sufficient length. It is also best not to compare writers of the opposite gender. Their styles show such discriminating properties that enable to make certain observations on the linguistic preferences of male and female authors, which would cloud the objectives of experiments.

Taking all these factors into account, two data sets were constructed, one comparing a pair of male writers, namely Jack London and James Curwood, and the second for a pair of female writers, Mary Johnston and Edith Wharton. The chosen writers are known for several sufficiently long novels, which were divided into groups to provide a base for training, and evaluation and test sets. The selection of studied texts is given in Table 1.

**Table 1:** The selection of literary works used in the research.

Training			
E. Wharton	The fruit of the tree	J. London	Martin Eden
	The custom of the country		Michael, brother of Jerry
	The house of mirth		Smoke Bellew
	The valley of decision		A daughter of the snows
M. Johnston	The long roll	J. Curwood	The hunted woman
	Audrey		Nomads of the North
	By the order of the company		Kazan
	Lewis Rand		God’s country—And the woman
Test 1			
E. Wharton	The age of innocence	J. London	The sea wolf
	The reef		The little lady of the big house
	The glimpses of the moon		The jacket
M. Johnston	To have and to hold	J. Curwood	Flower of the North
	Prisoners of hope		The valley of silent men
	1492		The flaming forest
Test 2			
E. Wharton	Summer	J. London	Burning daylight
	Ethan Frome		The mutiny of the Elsinore
	Bunner Sisters		The valley of the moon
M. Johnston	Sir Mortimer	J. Curwood	The country beyond
	Pioneers of the old South		The Alaskan
	Foes		The courage of Marge O’Doone

All analysed works were partitioned into much smaller text samples, with a comparable size of around 2 thousand words. For the training sets 25 text chunks were chosen per each of four novels per author, and for two test sets 15 samples per each of three novels per writer. It resulted in constructing the training sets including 200 samples, and two test sets with 90 samples each. For all text samples some statistics were calculated. They reflected the frequencies of occurrence for a group of common function words and punctuation marks, which have proven to be good style discriminators [41, 42].

**Fig 2:**
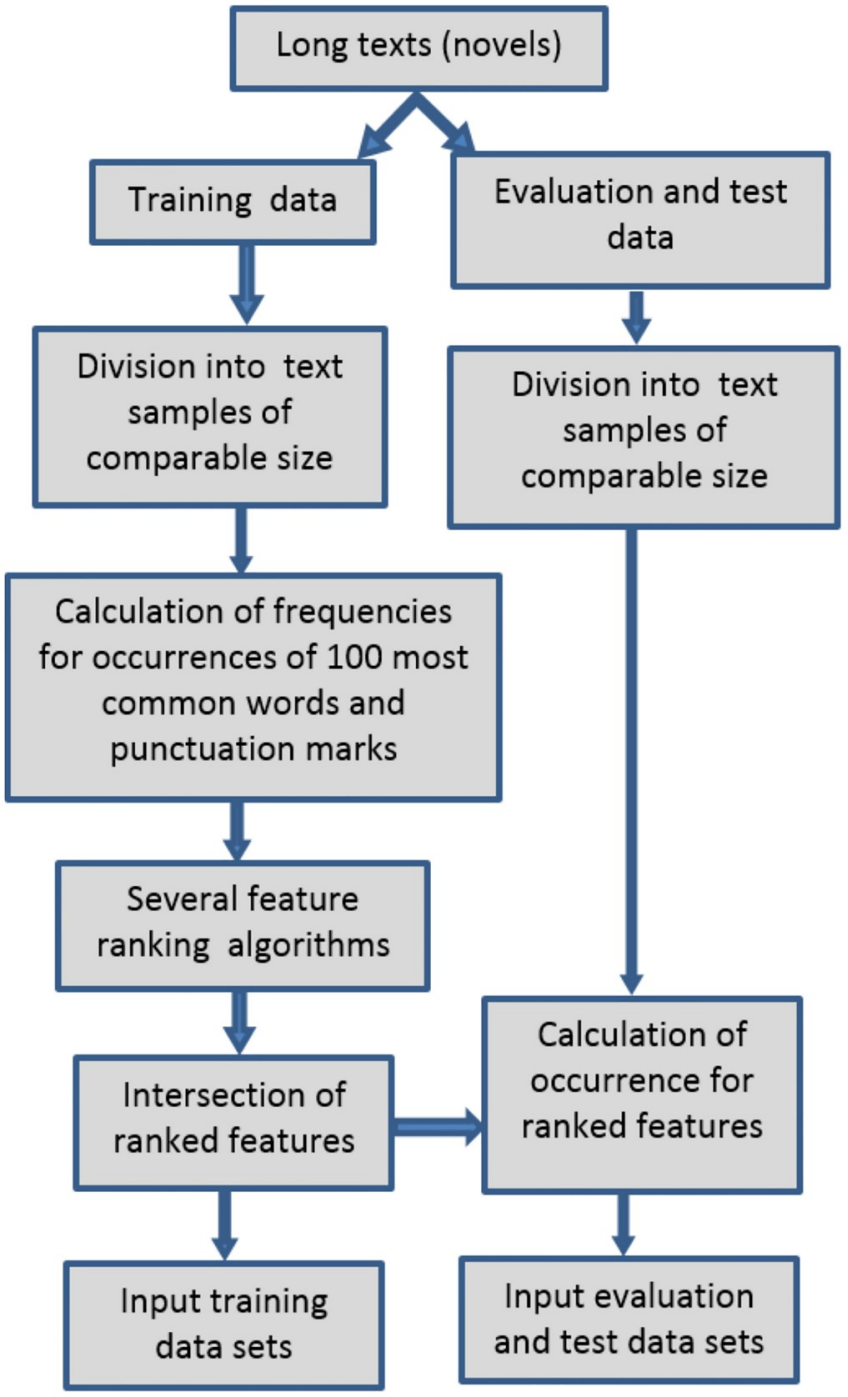
The employed procedure of input data preparation.

Function words were selected in the following manner. Referring to the list of the most frequently used words in the English language, firstly for the most common one hundred entries the frequencies were calculated for text chunks designated as a base for the training sets. To these variables the frequencies of regular punctuation marks were added. Then several feature ranking algorithms were employed to the data. For further processing only these attributes were chosen that were never considered as irrelevant. In other words, the set of the selected characteristic features consisted of variables included in the intersection of all rankings. The entire procedure is illustrated in the diagram shown in Fig 2.

This feature selection process resulted in the set of 24 attributes, including 22 lexical in nature (that, on, but, by, what, who, there, how, then, any, after, never, same, such, during, before, though, until, almost, whether, around, within), and 2 syntactic (semicolon, comma). The minimum value of any attribute equalled zero (when this attribute was absent in a text sample). The maximum in theory would be equal one, but only in the case when a text sample would contain just this feature (no other word, no punctuation marks), which in practice is next to impossible. So the obtained values of the attributes where always in the range <0, 1). Table 2 lists some statistical characteristics of these features for the training sets, for both male and female data sets.

**Table 2:** Statistical characteristics of attributes in the training sets.

Female writers	Feature	Male writers
Average ± Stand.dev.		Average ± Stand.dev.
0.01105107 ± 0.003442	that	0.01317706 ± 0.004606
0.00543583 ± 0.002661	on	0.00673523 ± 0.002870
0.00588084 ± 0.001974	but	0.00535185 ± 0.002247
0.00369755 ± 0.001963	by	0.00278382 ± 0.001549
0.00204511 ± 0.001322	what	0.00211854 ± 0.001302
0.00224227 ± 0.001497	who	0.00152172 ± 0.001078
0.00295941 ± 0.001644	there	0.00339445 ± 0.001875
0.00094113 ± 0.000809	how	0.00077539 ± 0.000853
0.00192844 ± 0.001282	then	0.00231427 ± 0.001345
0.00093867 ± 0.000811	any	0.00061570 ± 0.000653
0.00105569 ± 0.000784	after	0.00121316 ± 0.000934
0.00098878 ± 0.000863	never	0.00125856 ± 0.001074
0.00040399 ± 0.000458	same	0.00056841 ± 0.000617
0.00110639 ± 0.001085	such	0.00057576 ± 0.000703
0.00011204 ± 0.000283	during	0.00018787 ± 0.000346
0.00144544 ± 0.000946	before	0.00137534 ± 0.000834
0.00075369 ± 0.000700	though	0.00054694 ± 0.000685
0.00027586 ± 0.000484	until	0.00088509 ± 0.000867
0.00031832 ± 0.000451	almost	0.00066813 ± 0.000668
0.00010454 ± 0.000257	whether	0.00009506 ± 0.000233
0.00021629 ± 0.000400	around	0.00035876 ± 0.000510
0.00031865 ± 0.000444	within	0.00021422 ± 0.000363
0.00682859 ± 0.003382	;	0.00246340 ± 0.002065
0.07370084 ± 0.018293	.	0.06379152 ± 0.015922

Thus constructed input data sets were examples of binary classification with balanced classes, with continuous condition attributes and nominal class labels. From the point of recognition, both classes were considered to be of the same importance.

### Induction of decision rules from continuous data

Dominance-Based Rough Set Approach requires definitions of preference orders for values of all available attributes. In the case of continuous-valued features, recognised in the considered task, the ordering is natural, but preferences unclear. Also, as class labels are nominal and no author is preferred to any other, there is no visible ordering of values for the decision attribute. Defining preferences for criteria means creating associations between the calculated values of condition attributes and certain authors, reflecting characteristics of their writing styles. This information is not necessarily immediately accessible.

When preference orders cannot be directly implied from the available domain knowledge, they can be discovered through some auxiliary transformations [30], but such process is very time-consuming and computationally complex. To avoid it, a much simpler way was adapted. As only two class labels were considered, a single order was assumed for both data sets. For all condition attributes the same preference order was assigned, and tested for both “cost” and “gain” types, for minimal cover algorithms (they are referred to as MinC algorithms later in the paper). The induced rule sets were compared with respect to processing time, the number of inferred rules, and their quality evidenced by support. Even though in the case of minimal cover algorithms generation of rules is usually a matter of seconds, the time factor becomes important with repeated induction, also, it was reasonable to expect that the exhaustive algorithms would require much more time.

For both data sets the process of generation for one MinC algorithm took more time, and the rule set included more, but weaker rules, while the other showed much better characteristics in all these considered elements. These preference orders were selected that led to these better decision algorithms, gain type in the case of male writer data set, and cost type for female writer data set.

With the preference orders assigned to all criteria for male and female writers, all rules on examples were then induced in the exhaustive search (this algorithm is denoted as Exh later in the paper). As expected, the process of rule induction took a significant amount of time, in fact it took several days for each rule set, as thousands of decision rules were inferred, each including real-valued conditions on some attributes.

Next, both types of rule classifiers (MinC and Exh) were applied to the data. In the case of conflicts, a simple voting strategy was employed, giving each rule a single vote. As a measure of performance classification accuracy was used, understood as a ratio of correctly classified examples to the total number of examples, presented as percentage. Classification accuracy was a correct choice for a score of performance evaluation in the presented case since in all tasks classification was binary and classes balanced, and both classes were considered to be of the same importance. It means that the cost of false positives always equalled the cost of false negatives [11].

Firstly, constructed inducers were used for re-classification of learning samples. This step enabled to impose some additional hard constraints on decision rules. In each case such set was chosen that included rules with supports at least equal the threshold that was the highest of these that guaranteed the maximum classification accuracy for the learning samples. This approach was used for all further processing, for all executed experiments.

The constrained decision algorithms were evaluated by classifying samples from two test sets in the next step, and the results were selected as the reference points for comparison in further analysis. From all obtained results average values were calculated, and these are given in Table 3.

**Table 3:** Averaged performance of rule classifiers in continuous domain [%].

	Exh	MinC
Female writers	92.22	59.68
Male writers	93.33	53.51

The low performance of minimal cover decision algorithms partially resulted from imperfect coverage for test sets (on average it was 85.55% for female writers, and 79.45% for male). While minimal cover is generated, obviously it relates to learning samples and patterns discovered among them, which can be expressed and classified by some small number of rules. Yet in continuous domain conditions included in rules work against the probability of detecting the same detailed patterns in test data, which was confirmed by obtained results. With much higher numbers of rules provided by exhaustive algorithms also stronger rules were found, giving satisfactory predictions and coverage at 100%.

### Discretisation of the input data sets

In the presented research works to the training input data sets eight discretisation approaches were applied. They included:

- unsupervised
  - equal width binning—with varying the input parameter corresponding to the number of constructed bins: from 2 to 9 with a step of 1, from 10 to 90 with a step of 10, from 100 to 900 with a step of 100, from 1000 to 9000 with the step of 1000, and 10000 (36 variants)
    * in a standard approach (denoted as duwb),
    * with additional optimisation of obtained bins which led to their minimisation (denoted as duwo),
  - equal frequency binning
    * in a standard approach (denoted as duf)—with varying the input parameter that corresponded to the number of required bins from 2 to 200 with the step of 1 (199 variants)
    * in an approach with weighting bins (denoted as dufw)—with varying the input parameter, giving the required number of occurrences included in bins, from 1 to 200 with the step of 1 (200 variants)
- supervised (a single variant for each set)
  Fayyad and Irani
    * in a standard approach (denoted as dsF),
    * with additional optimisation of obtained bins which led to their minimisation (denoted as dsFo),
  Kononenko
    * in a standard approach (denoted as dsK),
    * with additional optimisation of obtained bins which led to their minimisation (denoted as dsKo).

Discretisation of tests sets was executed in two ways: independently on the learning samples, and by referring to ranges defined for the learning instances. Since the numbers of samples included in the test sets were different than in the training sets, in independent processing not all previously listed variants of input parameters were valid for them. For equal width binning the same parameters were used, and for non-parametric supervised methods the approach was the same.

In the case of equal frequency binning in the standard version the numbers of bins were varied from 2 to 90 with the step of 1 (89 variants), and from 1 to 90 with the step of 1 (90 variants) for the version with requirements on numbers of occurrences in bins. Then, in the executed tests such variants of training and test input sets were matched that were characterised by the same numbers of bins defined for the corresponding attributes. If the exact match was impossible to find, the closest variants were selected from the available alternatives.

For simplicity of representation and later processing, instead of using the intervals defined as the ranges specified by the established cut-points, all intervals were simply enumerated. Such positive integer numbers were next stored and used as attributes’ values, which allowed for a significant reduction of size in discretised rule sets.

### Discretisation of conditions in decision rules

Sets of inferred decision rules allow for a direct access to knowledge discovered in mining of data, given as conditions on attributes. Thus, the decision algorithms, induced previously from the continuous data, were discretised for each obtained variant of the discretised training sets. Discretisation was performed in the following manner.

For all attributes included in the premise part of a rule, a condition specified was matched with a certain interval to which the continuous value was assigned. Then the value in the condition was replaced with the selected interval, using its representation by an integer number.

As the result of such processing groups were constructed that consisted of the following elements: the discrete training sets, the decision algorithms with the conditions discretised using intervals defined for the training sets, and the test sets with as close matching of intervals as it was possible to find. The number of such groups was equal to the number of variants of the discrete learning data sets.

### Performance evaluation for discretised classifiers

Each decision algorithm, translated from the continuous into discrete domain as described above, was then employed for re-classification of the corresponding discretised learning set, and two test sets. Firstly, there was found the highest value of the minimal support required of rules that led to the maximum classification accuracy of the training samples. With this hard constraint on rules, the results for test sets were obtained.

For both male and female data sets, for each matching set of elements, two discrete decision algorithms (exhaustive and minimal cover) and two test sets, minimum, maximum, and average classification accuracies were found. There was also noted the coverage averaged for test sets, and obtained reduction of the rule set size, compared with respect to this size in continuous domain. These characteristics were studied separately for both types of decision algorithms, i.e. MinC and Exh. The experimental results are commented in the next section of the paper.

### Induction of decision rules from discrete data

Once the input data sets were discretised, it became possible to apply to them such data mining techniques that require discrete attributes, in particular decision rule induction methods. For the sake of comparison, once again rough set approach was considered. With the availability of categorical features DRSA could be replaced with CRSA, however, for both ways of processing inferring rules by exhaustive search was unfeasible due to the high number of prepared discrete variants of the training sets. In discrete domain rule induction is significantly simplified, yet it can still take several hours, as long as a day, and there were required multitudes of such processing time, hundreds. Thus the only type of algorithm, generation of which could be executed with some reasonable time constraints, was minimal cover.

Rough Set Exploration System (RSES) [61], dedicated to CRSA, includes implementation of LEM2 minimal cover algorithm. However, for discrete data it returns so few decision rules that coverage of test sets is often close to zero, which results in unacceptably low classification accuracy. These observations led to induction of minimal cover algorithm with DRSA, and MODLEM algorithm implemented in 4eMka software, the same that was used for induction of rules in continuous domain. The newly generated rule sets were once again applied as decision algorithms for re-classification of the training samples, and then their performance was evaluated with test samples.

## Observations on research results

The experimental results presented can be divided into three parts. The first was dedicated to performance of the discretised decision algorithms, for all considered variants of the discrete data sets. Unsupervised discretisation methods led to series of tests with varying the numbers of intervals defined for all attributes, while for supervised methods there were single tests. For all of them the observations are given for both types of decision algorithms generated (exhaustive and minimal cover), as different trends in performance were detected.

In the second part of given results some properties of the discretised rule sets were studied. The third group of results consisted of scores, obtained in evaluation of performance for minimal cover decision algorithms induced from the discrete input data sets.

### Unsupervised discretisation

Discretisation by construction of intervals with equal width is often criticised, as disregarding important data characteristics such as distribution of occurring values. When only minimum and maximum values are detected, and so defined range divided equally, in return some intervals can be established for which there were absolutely no occurrences of values in the considered sets. Nevertheless, since the number of bins is the input parameter, the procedures enforce the same numbers of intervals when applied to separate sets, which makes operations on them much simpler.

**Fig 3:**
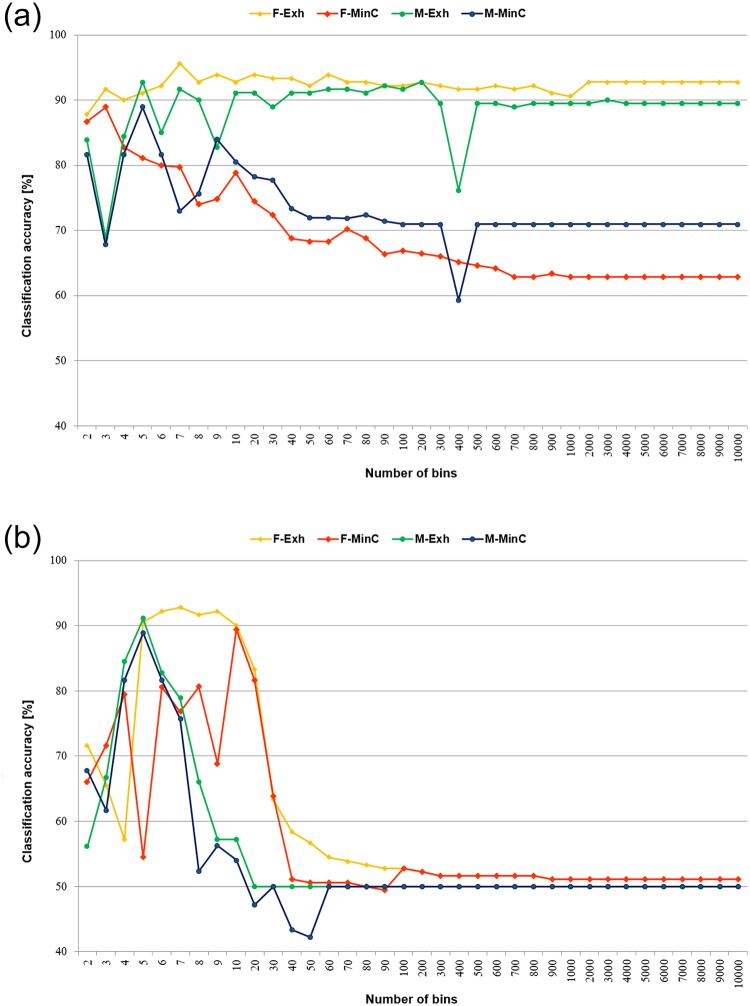
Averaged performance of rule classifiers in relation to the number of constructed intervals for unsupervised discretisation. A: Equal width binning (duwb). B: Optimised equal width binning (duwo).

The classification results for minimal cover (MinC) and all rules on examples (Exh) decision algorithms are displayed in Fig 3. The charts show the performance averaged over the test sets for each specified number of intervals constructed. For the exhaustive algorithms it can be observed that for the standard version of the discretisation method (duwb), actually in the whole range of the tested numbers of bins the performance can be considered as satisfactory.

For optimised equal width binning (duwo), the results were noticeably worse. For the relatively small numbers of bins the performance was to some extent acceptable, but for the higher numbers it was degraded too much. This was partially caused by the locality of the executed optimisation—since all sets were discretised independently, each was optimised independently as well. In turn, it caused much greater differences between the intervals defined for the training and test sets. And these differences resulted in the increased numbers of incorrect classifications of examples.

Due to the relatively poor performance of the minimal cover algorithms in continuous domain, caused by lower coverage, in discrete domain their observed power was increased for more variants of the discretised sets. The overall performance showed trends similar to those of exhaustive algorithms, i.e. satisfactory results for lower numbers of bins, and degraded for higher.

When the frequencies of occurrence of values are taken into account, such parts of the input continuous space, which are characterised by the presence of many points, are divided into many small and closely packed intervals. Other regions, with sparsely occurring values, have much fewer, yet larger bins. Thus this kind of processing more closely follows the distributions of input data points, and still, at least for some range, returns the same numbers of bins for independently discretised sets. Unequal numbers of constructed intervals can be caused by different cardinalities of sets with samples. Typically, the training sets include more examples than the evaluation and test sets. In such cases any attempts at enforcing the same numbers of bins are doomed to failure.

A modification of the standard equal frequency binning assigns some weights to intervals, where it is possible to define preferences for some regions of the feature space. When all bins (all regions) are treated in the same way, weighting them can be seen as a direct definition of the number of occurrences required for each interval. Hence in the standard version of discretisation algorithm (duf) a user specifies the numbers of bins, and the procedure ensures that in all intervals the same numbers of occurrences are included. For equal frequency binning with weights (dufw), the user defines the numbers of occurrences. Using this input parameter the target numbers of constructed intervals are established.

**Fig 4:**
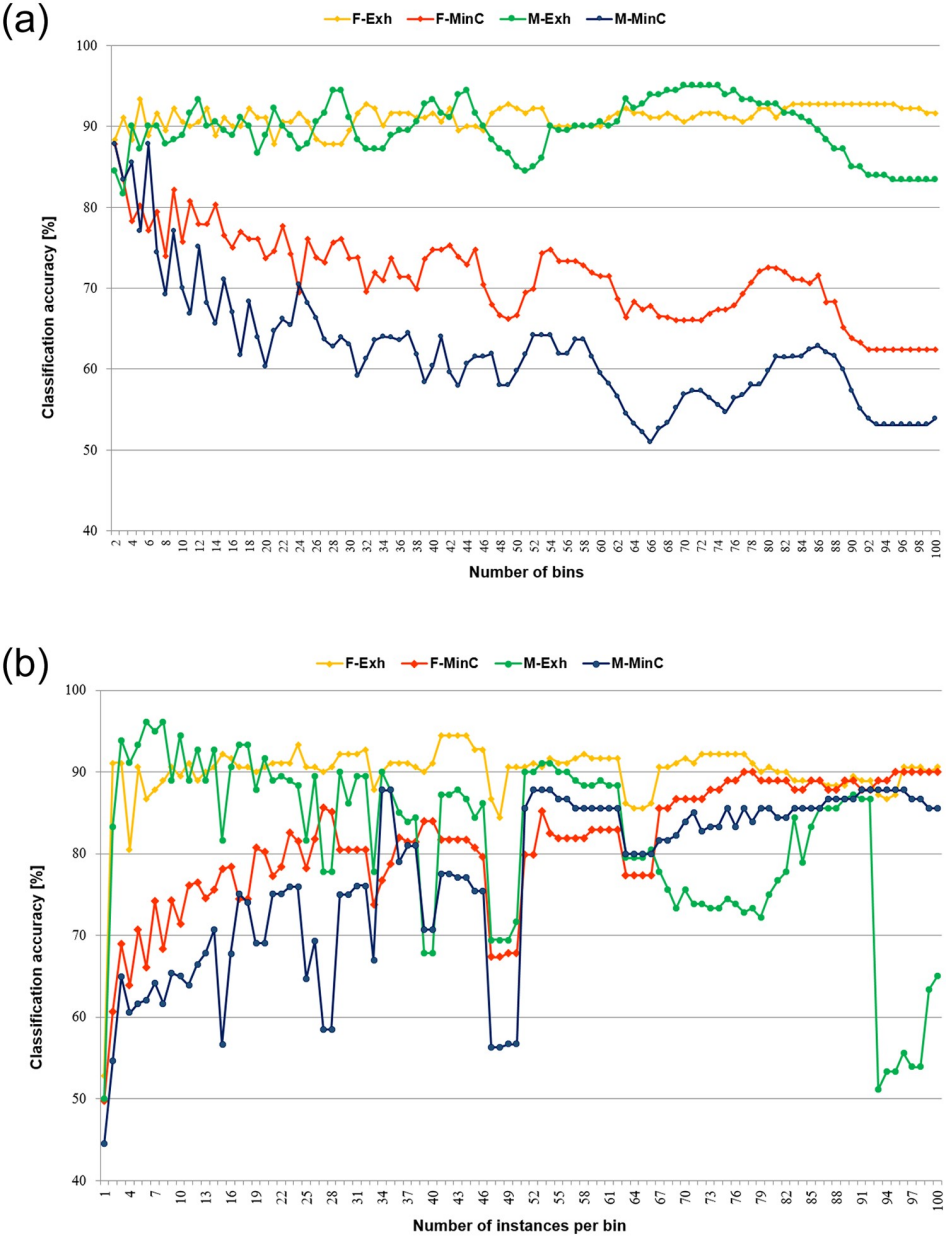
Averaged performance of rule classifiers for unsupervised discretisation. A: Equal frequency binning (duf). B: Equal frequency binning with weights (dufw).

For both MinC and Exh versions of the decision algorithm the classification results are displayed in Fig 4. The charts show the observed performance for the first half of the tested variants of the learning sets. For the second half the classification was very poor due to too large differences between the numbers of intervals for the training and test sets, resulting from independent discretisation of all sets.

For the exhaustive decision algorithms discretised by the standard equal frequency binning, almost in the whole presented range the classification accuracy was satisfactory, in several cases exceeding the reference levels of real-valued algorithms. The modification with interval weighting brought the worsening of the average performance, even though some of the obtained maxima were greater than the ones detected for the standard version of discretisation algorithm.

For minimal cover decision algorithms, the charts showed seemingly opposite trends in performance for the two versions of the discretisation method. This came from the fact that the trend in classification accuracy was related to the numbers of intervals, which in the standard version increased with the input parameter, and decreased for the weighted version. In both cases the highest correct prediction ratio could be found for the relatively low numbers of bins established.

### Supervised discretisation

Supervised discretisation is commonly praised for preserving the discriminating properties of values, occurring in the input sets, so intuitively we would expect better results from its application, than for the unsupervised approaches. In the tested cases, however, these supervised methods brought rather disappointing results, in particular for the exhaustive decision algorithms. For both types of algorithms, for Fayaad and Irani, and for Konnonenko method, the classification accuracies are shown in Table 4. For the minimal cover algorithm, since it initially performed so poorly, still some improvement could be observed. For the exhaustive algorithm the power was degraded, and not due to low coverage, but mainly because of the low level of minimum classification accuracy, in particular for male writer data set.

**Table 4:** Averaged performance of rule classifiers for supervised discretisation [%].

	Standard method		With optimised encoding	
	Exh	MinC	Exh	MinC
	Fayyad and Irani			
Female writers	90.56	78.34	87.78	75.56
Male writers	50.00	50.00	50.00	50.50
	Kononenko			
Female writers	88.89	80.21	88.89	83.89
Male writers	65.56	50.00	65.56	50.00

For the second version of Fayaad and Irani method, with optimised encoding, for both types of decision algorithms the observed performance was the same for male writers, and for female it was worse. For Kononenko approach, for all rules on examples for both versions of the discretisation method the results were the same, and for minimal cover some slight improvement could be detected for female writer data set.

These counterintuitive lower predictive properties of the discretised decision algorithms, in particular low minima, could be explained to some extent by a close adjustment of the constructed data models to the local context, that is to the particular sets. As they were discretised independently, all calculations and stopping criteria were based on the values occurring in each set. As not only the values but also the numbers of samples included in the sets were different, discretisation resulted in different definitions of intervals, and even in the varying numbers of those intervals for the same attributes in different sets. There were even cases that in one set for all values of some attribute the single interval was assigned, whereas in another set for the same attribute several bins were established.

To ensure that in all data sets the transformations of the same attribute were uniform, another attitude to discretisation of the evaluation and test sets could be employed—they could be discretised using the data models obtained for the training samples. Then the values present in the evaluation and test data sets were seen through the perspective of those values which occur in the learning set. The results from such processing are shown in Table 5.

**Table 5:** Averaged performance of rule classifiers for supervised discretisation with transformations of the test sets based on discrete data models obtained for the training samples [%].

	Standard method		With optimised encoding	
	Exh	MinC	Exh	MinC
	Fayyad and Irani			
Female writers	90.00	88.33	90.00	85.21
Male writers	91.11	80.67	90.56	84.45
	Kononenko			
Female writers	90.00	85.21	90.56	86.67
Male writers	90.56	84.45	90.56	84.45

The decision algorithms used in those experiments were the same as the ones obtained before, but they were applied to the modified test sets. It could easily be observed that this modified processing of some sets brought the expected improvement when compared to the previously shown results for supervised discretisation methods. In particular the minimum classification accuracies were significantly higher, which gave also much improved averaged performance for both types of decision algorithms.

### Properties of discretised rule sets

As in the presented case the recognised classes were considered to be of the same importance, the cost of false positive was considered to be equal to false negative. Therefore, the performance of the tested decision algorithms could be evaluated by specification of the number of correctly labelled samples in relation to the total number of samples, which results in the global classification accuracy, commented before in Sec. Unsupervised discretisation and Supervised discretisation. However, a study of coverage encountered for the test sets gave yet another insight, and enhanced understanding of the detected patterns, which were discretised by transformations of decision algorithms.

In the case of exhaustive algorithms, for all versions of discretised sets the coverage was always at 100%, and none of the test samples remained uncovered, so the returned classification accuracy resulted only from the correct and incorrect classifications, for all discretisation methods employed in the research.

**Fig 5:**
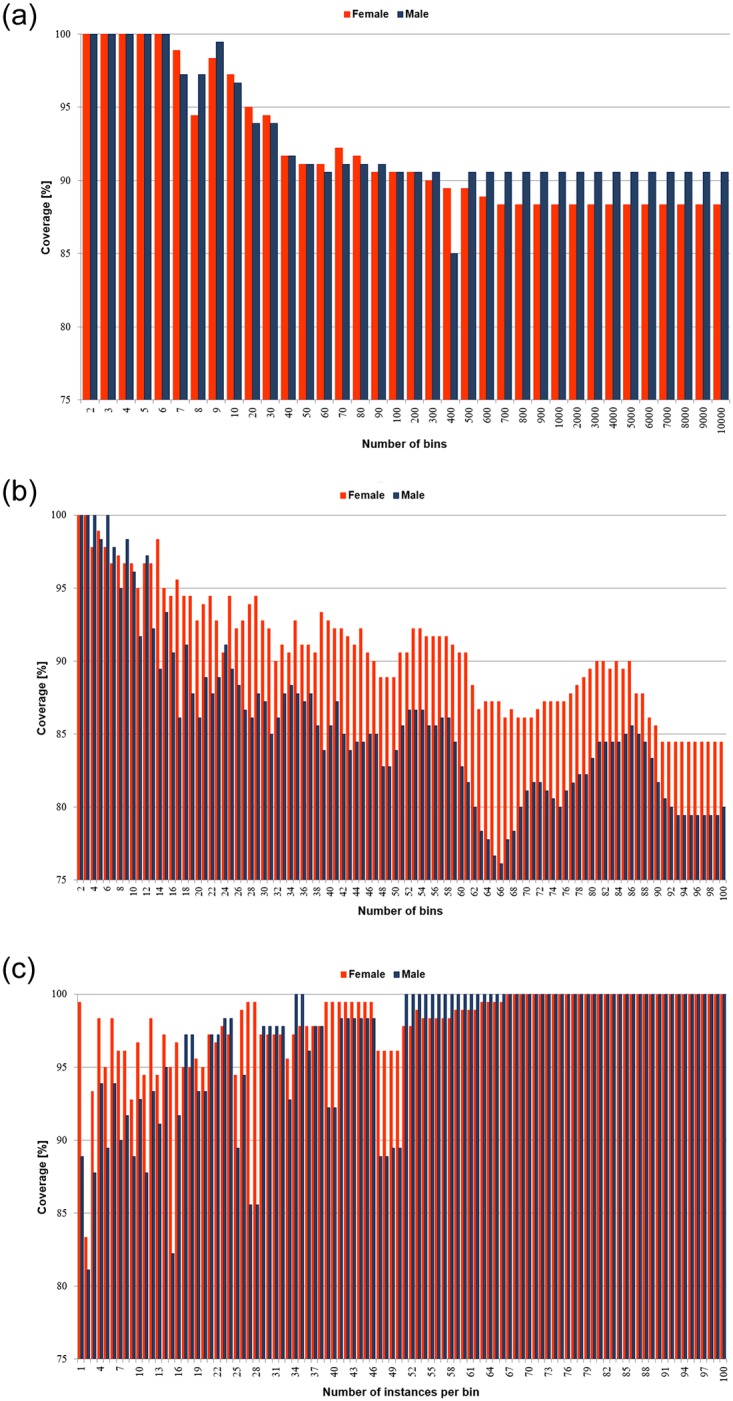
Averaged coverage of the minimal cover rule classifiers for unsupervised discretisation. A: Equal width binning (duwb). B: Equal frequency binning (duf). C: Equal frequency binning with weights (dufw).

For MinC algorithms the situation was markedly different, and the coverage highly dependent on the applied discretisation procedures and their parameters. From the four unsupervised discretisation methods, for equal width binning with optimised encoding the coverage was next to perfect—only for 3 and 4 numbers of bins, respectively 7-9 for female and 7-10 for male writers, there were some uncovered samples in the test sets. For the other three methods there were many more such cases, thus the charts presenting coverage are given in Fig 5.

It could be observed that for both versions of equal width binning and the standard equal frequency binning, for both data sets coverage was the best for the small numbers of intervals constructed, and then it gradually decreased while the numbers of intervals increased. Also, on average for equal width method for male writers the coverage was higher than for female writers, when the opposite could be stated for equal frequency binning. For equal frequency binning with weights, for both data sets at the beginning the numbers of intervals were high, hence low coverage, increased with the higher numbers of instances required per bins, which meant construction of fewer intervals.

For supervised discretisation approaches only in one case coverage was below 100%: for the standard Kononenko method, for female writer data set the coverage for MinC decision algorithm, averaged over test sets, equalled 98.89%.

**Fig 6:**
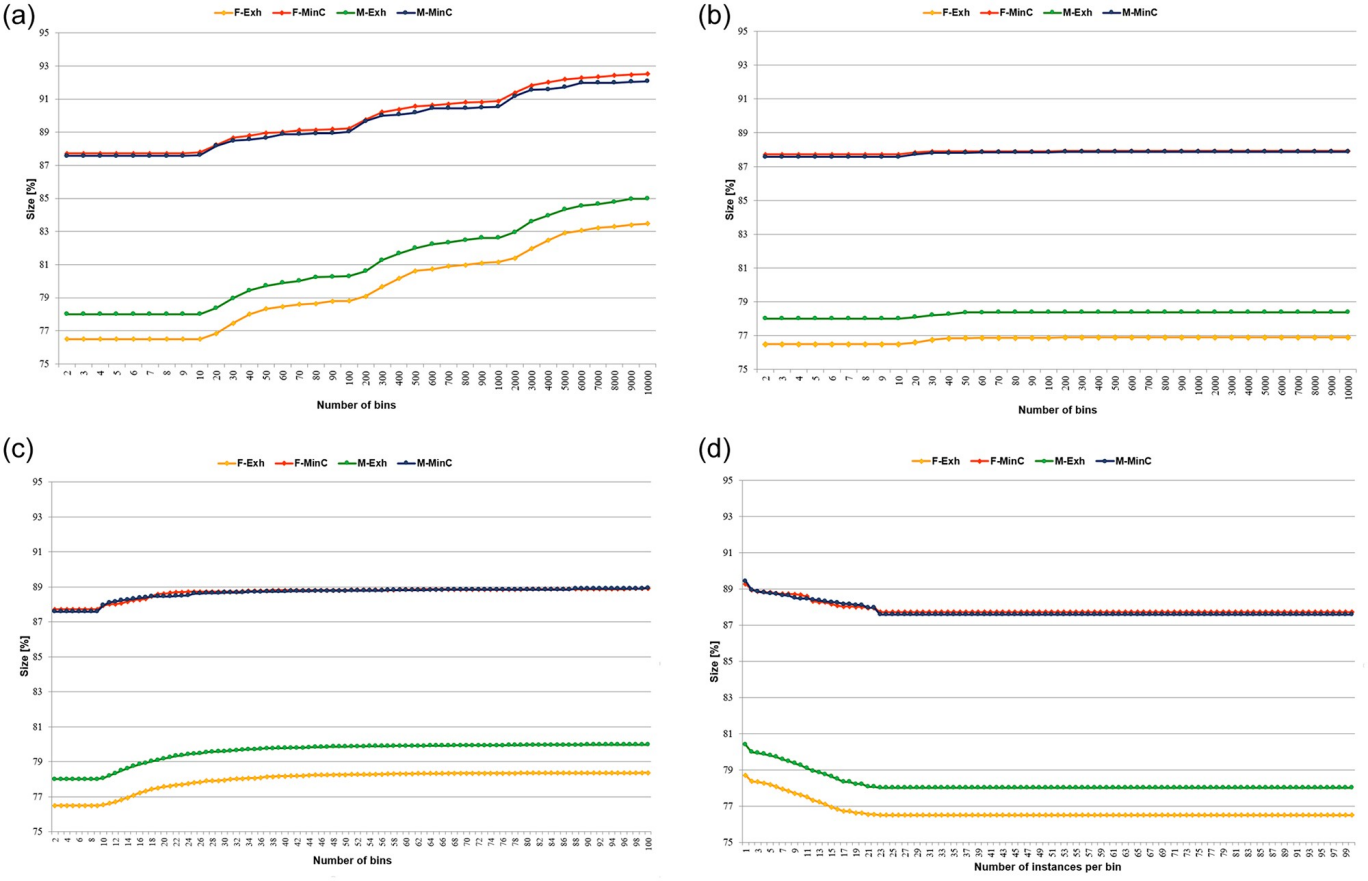
Reduction of size of rule sets for unsupervised discretisation. A: Equal width binning (duwb). B: Optimised equal width binning (duwo). C: Equal frequency binning (duf). D: Equal frequency binning with weights (dufw).

In discretisation of the induced rule sets, continuous values of conditions, included in the rule premises, were replaced with the nominal representations of intervals constructed. In this manner the numerical values, which required several digits to be stored, were replaced with single characters. It caused a significant reduction of size for both types of decision algorithms, shown for all unsupervised discretisation methods in Fig 6, and for supervised in Table 6.

**Table 6:** Reduction of size of rule sets for supervised discretisation [%].

	Exh	MinC
Female writers	76.50	87.71
Male writers	78.01	87.58

For minimal cover rule sets, as they were smaller, contained fewer rules and through that fewer conditions to be discretised, the size reduction was also smaller than in the case of exhaustive algorithms that included so many more rules. The results are given as the percentage presented by a size of a discretised algorithm with respect to the corresponding original size of decision algorithms, induced in continuous domain.

As it turned out, for both supervised discretisation methods employed in the research, and both their versions, for MinC and Exh algorithms the obtained reduction of size was the same, and the two differences observed were between two types of decision algorithms, and the two studied data sets.

### Induction of rules from discretised data

In the proposed methodology discretisation follows data mining, whereas typically discretisation is considered to be a part of initial data pre-processing, after which data mining follows. To confront the two ways, new rules were induced from all versions of the discrete input data sets, by using minimal cover algorithm in DRSA approach.

Due to the high number of the prepared variants of the discrete data sets (several hundreds per data set), induction of rule sets in exhaustive search would be a task of unmanageable proportions, when taking into account the computational costs involved. It was one of the considered factors motivating the proposed research framework. Because of the employed heuristics, minimal cover algorithms were found relatively quickly (in a matter of seconds), thus even if the generation procedure was repeated many times, still the task was feasible.

**Fig 7:**
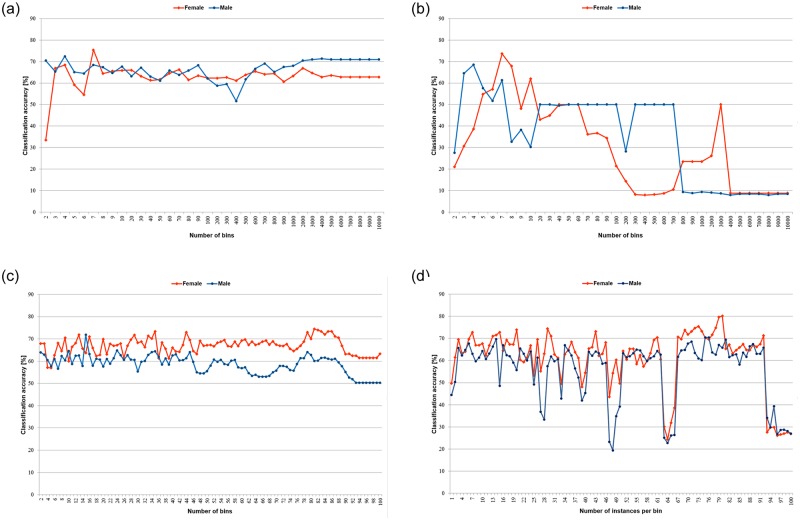
Averaged performance of the minimal cover rule classifiers induced from discrete data sets. A: Equal width binning (duwb). B: Optimised equal width binning (duwo). C: Equal frequency binning (duf). D: Equal frequency binning with weights (dufw).

Once all rules were inferred, they were applied for re-classification of the training and test samples, with simple voting strategy employed in case of conflicts, as in the earlier batch of tests concerning discretised rule sets. For all unsupervised discretisation methods, the average classification accuracy obtained for the test sets is shown in the charts included in Fig 7, and for supervised discretisation approaches in Table 7.

**Table 7:** Average performance of minimal cover rule classifiers induced from discrete input data for supervised discretisation [%].

	Standard method		With optimised encoding	
	Fayyad	Kononenko	Fayyad	Kononenko
Female writers	74.65	59.45	57.23	58.89
Male writers	36.54	40.36	45.63	40.36

When these results were compared against MinC decision algorithms working in continuous domain, it could be observed that for female writer data set only for optimised equal width binning the performance was worse, while for other discretisation methods on average it was improved. For male writers also for supervised discretisation the results were degraded. On the other hand, in all cases the discretised decision algorithms achieved better predictive accuracy than the rule sets induced from discrete data.

Nevertheless, it is not entirely out of the question that for some variant of the discrete input data set an algorithm induced in exhaustive search could outperform both types of decision algorithms with continuous values, and with the discretised conditions. How to find out which particular approach to discretisation would cause such advantageous conditions remains the open problem.

### Summary of the obtained results

For all tested variants of decision algorithms and input data sets Table 8 provides average classification accuracy and the calculated standard deviation. For simplicity of comparisons, there were also included the reference points of average performance for decision algorithms induced and operating in continuous domain.

**Table 8:** Summary of performance for all rule classifiers [%].

Decision algorithm	Contin. domain	Discretisation method				
		duwb	duwo	duf	dufw	ds
F-Exh	92.22	92.38 ± 1.68	61.03 ± 15.37	91.15 ± 1.56	89.93 ± 04.41	89.03 ± 1.03
F-MinC	59.68	69.30 ± 7.71	58.14 ± 12.37	71.41 ± 5.59	81.12 ± 07.69	79.50 ± 4.83
M-Exh	93.33	88.64 ± 5.58	55.29 ± 11.58	89.69 ± 3.61	81.50 ± 11.11	57.78 ± 7.78
M-MinC	53.51	73.33 ± 5.52	54.24 ± 11.15	62.06 ± 7.57	77.09 ± 10.64	50.00 ± 0.00
F-DMinC		62.90 ± 6.71	30.77 ± 21.40	66.95 ± 4.63	61.06 ± 13.90	62.55 ± 7.21
M-DMinC		66.16 ± 5.41	35.18 ± 20.81	58.76 ± 4.71	55.86 ± 13.90	40.72 ± 7.55

For continuous domain the averages are listed, and for all discretisation methods overall average± standard deviation for algorithms discretised and induced from discrete data (denoted as DMinC).

The summary indicates that the widest ranges of power of rule classifiers, evidenced by the relatively high values of standard deviation, were detected for optimised equal width binning (duwo), and for equal frequency with weights (dufw). The lowest values of standard deviation were obtained for equal frequency binning, followed close by supervised discretisation. However, for the latter the accompanying predictive accuracies were mostly worse than for unsupervised methods.

As far as the average classification accuracies were concerned, for female authors and exhaustive algorithms only equal width binning (duwb) gave a slight improvement, while for other discretisation approaches the results were worse than for continuous domain. For male writers and Exh algorithms, for all methods on average classification was degraded with respect to considerations of real-valued conditions. On the other hand, for minimal cover algorithms, since their performance for continuous data was relatively poor, it was much easier to find some improvement.

Furthermore, it needs to be remembered that Table 8 lists only the overall averages, calculated over series of tests. In the previously presented detailed plots, in unsupervised methods there could be detected many values of the input parameter, which led to the significant improvements of performance for the discretised rule classifiers.

The results from experiments show the merit of the reversed attitude to discretisation of data. Instead of executing it as a part of initial pre-processing before data mining, we can mine data first. Then we discretise both data and constructed inducers, and still obtain satisfactory results. With such changed order, in the data mining phase we have access to all available information and do not risk any loss of it, which most often happens during discretisation. On the other hand, later translation from continuous into discrete domain helps to obtain more general and compact forms of constructed inducers, shortens processing time, while keeping or even improving predictions, and enables to observe results for many disretisation approaches at reasonable computational costs. These advantages of the proposed methodology should be weighted against some disadvantages, such as an increased complexity of discretisation, and a lack of guarantee that discretised rule sets perform better than some of decision algorithms which could be induced from discrete data.

## Concluding remarks

The paper presents the research on discretisation applied to the decision rules induced from the continuous data, thus changing the standard order of the processing steps. The proposed methodology relied on mining the complete available data, without any attempts at its initial simplification or reduction, which was then followed by such simplification of the learned models through their discretisation.

The research framework enabled to test various discretisation approaches with acceptable costs of processing, as the most computationally demanding stage of data mining and knowledge discovery was executed just once, and only discretisation was performed repeatedly. One of the disadvantages of such methodology could be found in more complex discretisation, since not only data sets but also learned models were discretised. Also, no guarantee could be given that a better performing inducer could not be found for some discrete version of data sets. On the other hand, for the typical order of processing, with discretisation preceding data mining, such guarantee neither could be given.

The real-valued input characteristic features, used in the research works, came from the application domain of stylometric analysis of texts. For the task of authorship attribution, writing styles were defined by some quantitative descriptors of lexical and syntactic type. They gave the frequencies of occurrence for the selected common function words and punctuation marks.

From the prepared continuous learning data sets decision rules were inferred within Dominance-Based Rough Set Approach, for two types of algorithms, minimal cover and exhaustive. The induced rules contained real-valued conditions that meant a very close fit to the training data, and detailed definitions of the concepts. By itself, it could be considered an advantage, but it could cause poorer generalisation, lengthen the classification process, and increase the requirements for data storage.

In the next stage of the experiments, all input data sets were discretised, using the selected unsupervised and supervised strategies, with varying their parameters. The previously constructed inducers were transformed as well, by substituting the continuous conditions on the attributes with their discrete forms. So translated rule classifiers were next applied for classification of samples in discretised sets.

The results from the performed tests show that for unsupervised discretisation approaches on average the inducers outperformed those constructed for supervised methods. For exhaustive algorithms some improvements in classification accuracies were detected, but for minimal cover algorithms the positive differences in performance were much higher. However, it should be remembered that the results of rule induction and classification depend on data, distributions of features’ values, both condition attributes and decision classes.

Some properties of the discretised rule sets were also studied. Coverage obtained for the test sets by the exhaustive algorithms was always perfect, but for minimal cover algorithms highly dependent on a discretisation strategy and its parameters. The sizes of the transformed rule sets were significantly reduced with respect to continuous domain, due to replacing the real-valued conditions with their categorical representations. For comparison sake, from discrete data sets minimal cover algorithms were also induced. Their performance, contrasted with decision algorithms with the real-valued and discretised conditions, turned out to be worse.

In the described research works the proposed approach was dedicated to rule classifiers, in which the access to the learned knowledge is relatively straightforward. In the future research, the methodology will be tested for other types of inducers capable of working on continuous attributes while allowing for easy access to their structures. An another research path will be directed at discretisation executed with regard to not the whole data, but only conditions present in the inducers constructed in continuous input space. It would allow to use even more knowledge, discovered in the data mining phase, for the processes dedicated to building the discrete data models. Also, the proposed methodology will be tested in other application domains, for other data sets.

## Supporting information

S1 File (ZIP)
